# Taste Detection Threshold in Relation to Pfizer COVID-19 Vaccine

**DOI:** 10.7759/cureus.33786

**Published:** 2023-01-15

**Authors:** Saja Q Abbas, Taghreed F Zaidan

**Affiliations:** 1 Department of Oral Diagnosis, College of Dentistry, University of Baghdad, Baghdad, IRQ; 2 Department of Dentistry / Oral Medicine, University of Al-Turath, Baghdad, IRQ

**Keywords:** taste detection threshold, side effects, health care workers, pfizer vaccine, covid-19

## Abstract

Background

The first hit of the COVID-19 pandemic was reported in December 2019 in Wuhan, Hubei province, China, and wholesale seafood markets were reported to be the source of infection. The development of effective and safe vaccines against SARS-CoV-2 has been extremely fast. The development of the COVID-19 mRNA vaccine started in early January 2020, after the release of the SARS-CoV-2 genetic sequence by the Chinese Center for Disease Control and Prevention and its global dissemination by the Global Initiative on Sharing All Influenza Data (GISAID).

Aim

The current study aims to evaluate the taste detection threshold after the second shot of Pfizer COVID-19 vaccination at different intervals and after the booster shot.

Materials and methods

A multistage sampling technique was used to select all Iraqi healthcare workers (HCWs) to whom the inclusion criteria were applied, from Baghdad Medical City and Al-Yarmouk Teaching Hospital participated in this study. The total number of selected subjects was 85. The ages of HCWs ranged from 24 to 60 years. The first study group (G1) comprised 30 HCWs who received the COVID-19 vaccination from Pfizer in two doses. The subjects were recruited into this group two weeks to three months after being vaccinated and had no history of COVID-19 symptoms. The second group (G2) comprised 30 HCWs who received the COVID-19 vaccination from Pfizer in two doses. They were recruited three to six months after they were vaccinated and had no prior history of the virus’ symptoms. The third group (G3) was of 25 HCWs who received a third booster dose of COVID-19 vaccination from Pfizer two weeks after the booster dose. The taste detection threshold was performed for four basic tastes: sweet, sour, salt, and bitter.

Results

The taste detection threshold for (sweets) showed a significant difference between the first and second groups and the second and third groups. The taste detection threshold of sweets was significantly higher in the second group.

Conclusions

After three to six months from getting the second dose, the taste detection threshold for sweets was assessed as high. This means that the flavor will be harder to perceive. Pfizer COVID-19 immunization may be assumed to be one of the causes of defected taste sensation to sweet flavor.

## Introduction

The acronym COVID-19 is made up of four different parts: “CO” refers to the corona, “VI” means “virus,” “D” means “disease,” and “19” refers to the year it was created (2019) [1]. In December 2019, Wuhan, China, became the site of the first report. It started in seafood markets [2]. At the seafood market, live creatures such as bats, rabbits, snakes, marmots, frogs, and birds are typically sold [3]. Severe acute respiratory syndrome Coronavirus 2 (SARS-CoV-2) is the name given to the virus by the International Committee on Taxonomy of Viruses (ICTV) [4].

During an emergency meeting held by the World Health Organization on January 30, 2020, COVID-19 was named an international public health emergency of international concern [5]. Since it was found in December 2019, the virus has quickly spread worldwide, causing chaos in all parts of society [6].

This pandemic’s presence was not unprecedented in history. The WHO noted that more than 8000 individuals were harmed, and 774 were killed in 2002-2003 due to the coronavirus known as SARS [7]. More than 2494 people were affected by the MERS-CoV outbreak in 2012, and more than 858 people died worldwide [8].

In Iraq, the first COVID-19 infections were reported in February 2020 in southern Iraq [9]. The action plan initiated early screening stations in hospitals, airports, and land ports. Until April 10, 2020, The Iraqi Ministry of Health has checked for 33889 possible illnesses. There were 1,318 cases in Iraq, 45.59% of whom survived, for a case fatality rate (CFR) of 5.46% [10].

On May 10, 2021, vaccinations against COVID-19 were introduced in Iraq. By July 5, 2021, 0.97% of Iraqis would have gotten all doses of the COVID-19 vaccine, and 1.74% would have received the first dose [11].

After receiving a vaccination, building immunity might occasionally have unfavorable side effects. The Pfizer-BioNTech COVID-19 vaccine may have mild general adverse effects after the first, second and third dose, including pain, redness and swelling at the injection site, fatigue, muscle and joint aches, headaches, fever, and chills. These symptoms may indicate that the body is building its defenses to defend itself [12].

In a study done in 2021, about facial symptoms, changes in sensitivity and paralysis were investigated. Among the oral symptoms assessed were burning sensations, pain, taste changes, oral aphthous-like lesions, xerostomia, tongue depapillation, stomatitis/mucositis, commissural cheilitis, and oral candidiasis. After the first and second dosages, 2.7% and 3.1% of the sample, respectively, reported alterations in taste sensitivity [13].

We aimed to evaluate the taste detection threshold after the second and third doses of Pfizer COVID-19 vaccination in Iraqi HCWs. To our knowledge, no previous study has evaluated the taste detection threshold after the Pfizer vaccine by chemosensory test.

## Materials and methods

Subjects

A multistage sampling technique was used to select all Iraqi HCWs to whom the inclusion criteria were applied, from Baghdad Medical City and Al-Yarmouk Teaching Hospital to enroll in the current study. The total number of selected subjects was 85. The ages of the workers ranged from 24 to 60 years. Healthcare workers were examined in these hospitals, and each person who participated in this study consented. The study period for recruitment was from January 2022 to April 2022.

A stratified sampling was used to divide the study subjects into three groups. Group 1 (G1) included all HCWs (30 HCWs) who received the Pfizer COVID-19 vaccination in two doses. They were recruited two weeks to three months after being vaccinated and had no prior history of the virus’ symptoms. While group 2 (G2) included all HCWs (30 HCWs) who received the Pfizer COVID-19 vaccination in two doses. They were recruited three to six months after being vaccinated and had no history of the virus’ symptoms. And group 3 (G3) included all HCWs (25 HCWs), who received a third (booster) dose of COVID-19 vaccination from Pfizer. They were recruited two weeks after the third dose.

Inclusion criteria: any Iraqi healthcare worker aged 24-60 years with no history of previous COVID-19 virus symptoms. Exclusion criteria: vaccinated subjects with a history of symptomatic COVID-19 virus infection, pregnant women, subjects who received two types of vaccine and subjects with any systemic disease.

This study was performed after receiving ethical approval from the Ethics Committee, College of Dentistry, University of Baghdad (Ref # 444, date: January 3, 2022).

Assessment of taste detection threshold

Each taste gradient was composed of 15 solutions ranging from 1.5 to 15.5 mmol in 1 mmol increment for sucrose, 1 to 78 mmol in 5.5 mmol increments for sodium chloride, 48 to 720 μmol in 48 μmol increments for citric acid, and 89 to 117 mmol (in 2 mmol increments) for urea, as shown in Table 1 [14].

**Table 1:** Different concentrations of sucrose, sodium, citric acid, and urea used

Concentration number	Sucrose, mmol/L (Sweet)	Sodium chloride, mmol/L (Salt)	Citric acid, μmol/L (Sour)	Urea, mmol/L (Bitter)
1	1.5	1	48	89
2	2.5	6.2	96	91
3	3.5	12	144	93
4	4.5	17.5	192	95
5	5.5	23	240	97
6	6.5	28.5	288	99
7	7.5	34	336	101
8	8.5	39.5	384	103
9	9.5	45	432	105
10	10.5	50.5	480	107
11	11.5	56	528	109
12	12.5	61.5	567	111
13	13.5	67	624	113
14	14.5	72.5	672	115
15	15.5	78	720	117

A volume of 10 ml of each taste gradient solution, brought to room temperature (22-25°C), was presented to participants by randomly-coded disposable cups. The taste cups were sequentially ordered from deionized water (zero flavor conc.) to higher concentrations [15]. The sip-and-spit technique was used, in which the tasting solution was briefly swirled in the mouth before being expectorated into an empty cup [16].

Statistical methods

Data analysis was performed using the available statistical tool SPSS version 28 (IBM Corp., Armonk, NY, USA). The information was shown using elementary statistical measures, including frequency, percentage, mean, standard deviation, and range (mini-max values).

The significance of differences between means was analyzed using either the Student’s t-test or the analysis of variance (ANOVA) test. The t-test was used to compare differences between two independent means, while the analysis of variance (ANOVA) test was used to compare differences between more than two independent means.

## Results

Demographic findings (age and gender)

For G1, their mean age was 32.8±9.5 years, ranging from 23 to 59 years. Nine men (30%) and 21 females (70%) were in this group. For G2, their mean age was 31.7±8.7 years, ranging from 23 to 55 years. Twenty-five subjects were female (83.3%), and five (16.7%) were male. For G3, their mean age was 30.8±4.5 years, ranging from 24 to 40 years. There were 21 females (70%) and nine males (30%).

ANOVA tests found no significant difference in age between the G1, G2, and G3 (p>0.05). A significant difference in the means of male and female subjects was discovered (P=0.019). There was a significant gender difference between G2 and G3 (P=0.005) by using the ANOVA test.

Taste detection threshold

Sweet

**Figure 1:**
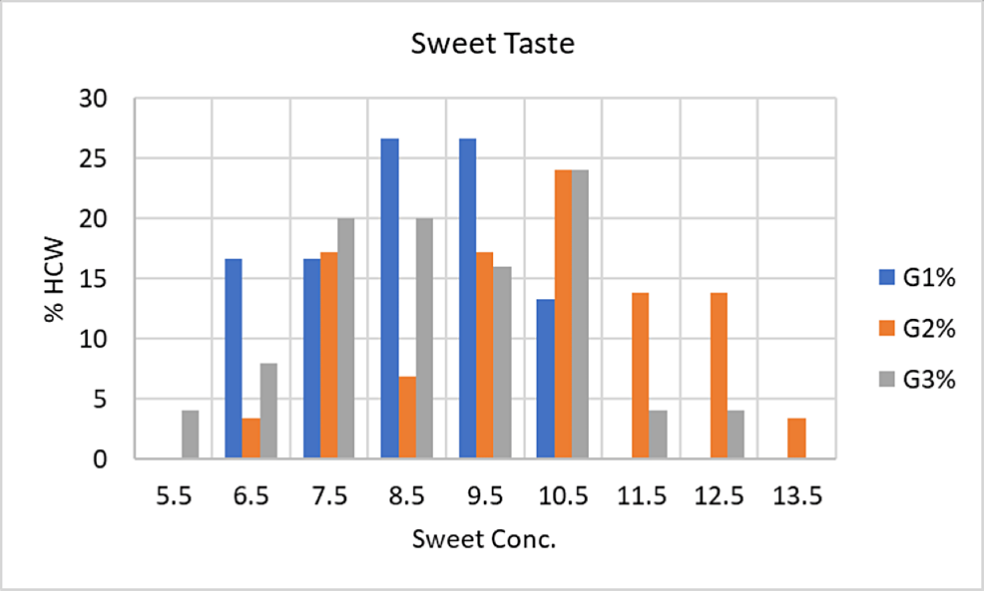
Clinical presentation of taste detection threshold (Sweet) of the three study groups G1: Group 1; G2: Group 2; G3: Group 3; HCW: Health care worker; Conc: concentration

The average concentration of sucrose detected by subjects in G1 was 8.53±1.30 mmol/L, with a range of 6.5 to 10.5 mmol/L. Five participants (16.7%) reported tasting sweetness at concentration number 6 (6.5 mmol/L). Five participants (16.7%) reported tasting sweetness at concentration number 7 (7.5 mmol/L). Eight participants (26.7%) reported tasting sweetness at concentration number 8 (8.5 mmol/L). Eight participants (26.7%) reported tasting sweetness at concentration number 9 (9.5 mmol/L). Finally, four participants (13.3%) tasted sweetness at concentration number 10 (10.5 mmol/L). These outcomes are illustrated in Figure 1.

The average amount of sucrose detected by subjects in the G2 was 10.17±1.94 mmol/L, with a range of 6.2 to 13.5 mmol/L. One participant (3.4%) reported tasting sweetness at concentration number 6 (6.5 mmol/L), five participants (17.2%) at concentration number 7 (7.5 mmol/L), and two participants (6.9%) at concentration number 8 (8.5 mmol/L). In addition, five participants (17.2%) reported tasting sweetness at concentration number 9 (9.5 mmol/L), seven participants (24.1%) at concentration number 10 (10.5 mmol/L), four participants (13.8%) at dosage number 11 (11.5 mmol/L), and one person (3.4%) at concentration number 13 (13.5 mmol/L). These results are presented in Figure 1.

The average amount of sucrose detected by subjects in G3 was 8.94±1.71 mmol/L, with a range of 5.5 to 12.5 mmol/L. One participant (4.0%) tasted sweetness at concentration number 5 (5.5 mmol/L), two participants (8.0%) at concentration number 6 (6.5 mmol/L), five participants (20.0%) at concentration number 7 (7.5 mmol/L), five participants (20.0%) at concentration number 8 (8.5 mmol/L). In addition, four participants (16.0%) tasted sweetness at concentration 9 (9.5 mmol/L), six participants (24.0%) at concentration 10 (10.5 mmol/L), one person (4.0%), at concentration 11 (11.5 mmol/L), and one participant (4.0%) at concentration 12 (12.5 mmol/L) (see Figure 1).

The taste detection thresholds for sweetness were significant between the means of the concentrations of the three groups (P = 0.001). The ANOVA test showed a significant difference between G1 and G2 (P = 0.001) and between G2 and G3 (P = 0.017). However, there was no significant difference between G1 and G3 (P = 0.321). Consequently, the taste detection threshold in the second group was higher than in the first and third groups.

Salt

**Figure 2:**
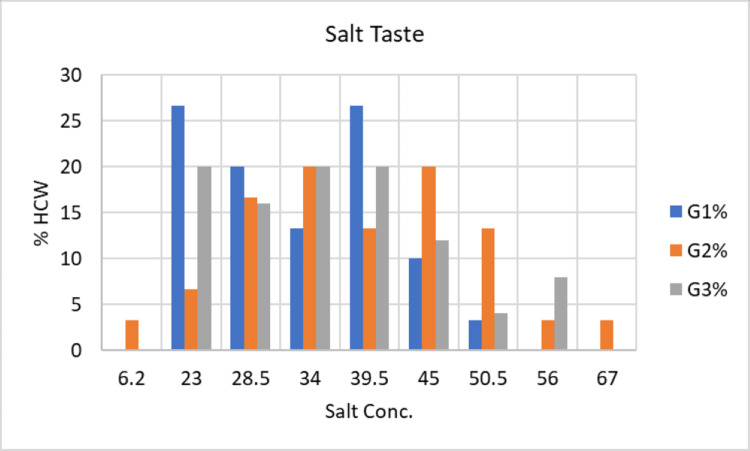
Clinical presentation of taste detection threshold (Salt) of the three study groups. G1: Group 1; G2: Group 2; G3: Group 3; HCW: Health care worker; Conc: concentration

The average sodium chloride concentration detected by subjects in G1 was 33.08±8.31 mmol/L, with a range of 23 to 50.5 mmol/L. Eight participants (26.7%) reported tasting salt at concentration number 5 (23 mmol/L), six participants (20.0%) at concentration number 6 (28.5 mmol/L), and four participants (13.3%) at concentration number 7 (34 mmol/L). In addition, eight participants (26.7%) reported tasting salt at concentration number 8 (39.5 mmol/L), three participants (10.0%) at concentration number 9 (45 mmol/L), and one participant (3.3%) at concentration number 10 (50.5 mmol/L), as shown in Figure 2.

The subjects in G2 detected a mean sodium chloride concentration of 38.39±11.89 mmol/L with a range of 6.2 to 67 mmol/L, according to the results. One participant (3.3%) had tasted salt at concentration number 2 (6.2 mmol/L), two participants (6.7%) at concentration number 5, and five participants (16.7%) at concentration number 6 (28.5 mmol/L). In addition, six participants (20.0%) reported tasting salt at concentration number 7 (34 mmol/L), four participants (13.3%) at concentration number 8 (39.5 mmol/L), and six participants (20.0%) at concentration number 9 (45 mmol/L). Furthermore, four participants (13.3%) reported tasting salt at concentration number 10 (50.5 mmol/L), one participant (3.3%) at concentration number 11 (56 mmol/L), and one participant (3.3%) at concentration number 13 (67 mmol/L), as presented in Figure 2.

The third group’s results revealed that the mean concentration of sodium chloride detected by subjects in G3 was 35.76±10.01 mmol/L and ranged from 23 to 56. Five participants (20.0%) tasted salt at concentration number 5 (23 mmol/L), four participants (16.0%) at concentration number 6 (28.5 mmol/L), five participants (20.0%) at concentration number 7 (34 mmol/L), and five participants (20.0%) at concentration number 8 (39.5 mmol/L). In addition, three participants (12.0%) reported tasting salt at concentration number 9 (45 mmol/L), one participant (4.0%) at concentration number 10 (50.5 mmol/L), and two participants (8.0%) at concentration number 11 (56 mmol/L) (see Figure 2).

The results reveal no significant difference between the means of the concentrations of the three groups for the taste detection threshold of salt (P = 0.137). The ANOVA test showed no significant difference between G1 and G2 (P = 0.094), G2 and G3 (P = 0.384) and between G1 and G3 (P = 0.283).

Sour 

**Figure 3:**
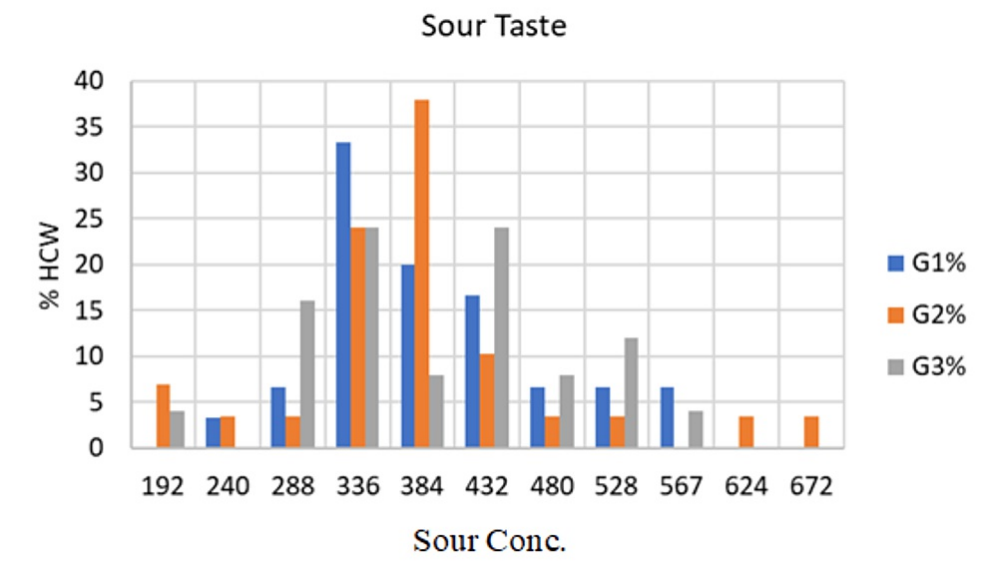
Clinical presentation of taste detection threshold (Sour) of the three study groups G1: Group 1; G2: Group 2; G3: Group 3; HCW: Health care worker; Conc: concentration

The results showed that the mean citric acid concentration detected by subjects in G1 was 393.0±82.67 μmol/L in the range from 240 to 567 μmol/L. One subject (3.3%) tasted sour at concentration number 5 (240 μmol /L), two subjects (6.7%) at concentration number 6 (288 μmol/L), 10 subjects (33.3%) at concentration number 7 (336 μmol/L), and six subjects (20.0%) at concentration number 8 (384 μmol/L). In addition, five subjects (16.7%) tasted sour at concentration number 9 (432 μmol/L), two subjects (6.7%) at concentration number 10 (480 μmol/L), two subjects (6.7%) at concentration number 11 (528 μmol/L), and two subjects (6.7%) at concentration number 12 (567 μmol/L), as presented in Figure 3.

The results showed that the mean concentration of citric acid detected by subjects in G2 was 392.0±114.20 μmol/L and ranged from 192 to 672 μmol/L. Two subjects (6.9%) tasted sour at concentration number 4 (192 μmol /L), one subject (3.4%) at concentration number 5 (240 μmol/L), one subject (3.4%) at concentration number 6 (288 μmol/L), seven subjects (24.1%) at concentration number 7 (336 μmol/L). In addition, 11 subjects (37.9%) tasted sour at concentration number 8 (384 μmol/L), three subjects (10.3%) at concentration number 9 (432 μmol /L), and one subject (3.4%) at concentration number 10 (480 μmol/L). Lastly, one subject (3.4%) tasted sour at concentration number 11 (528 μmol/L), one subject (3.4%) at concentration number 13 (624 μmol/L), and one subject (3.4%) at concentration number 14 (672 μmol/L), as seen in Figure 3.

The mean concentration of citric acid detected by subjects in G3 was 393.24±94.29 μmol/L and ranged from 192 to 567 μmol/L. One subject (4.0%) tasted sour at concentration number 4 (192 μmol/L), four subjects (16.0%) at concentration number 6 (288 μmol/L), and six subjects (24.0%) at concentration number 7 (336 μmol/L). In addition, two subjects (8.0%) tasted sour at concentration number 8 (384 μmol/L), six subjects (24.0%) at concentration number 9 (432 μmol/L), and two subjects (8.0%) at concentration number 10 (480 μmol/L). Finally, three subjects (12.0%) tasted sour at concentration number 11 (528 μmol/L) and one subject (4.0%) at concentration number 12 (567 μmol/L), as shown in Figure 3.

The taste detection threshold for sour exhibited no significant variation between the means of the concentrations of the three groups (P = 0.999), as illustrated in Figure 3. There was no discernible difference between G1 and G2 (P = 0.969), G2 and G3 (P = 0.966) and between G1 and G3 (P = 0.992) by using the ANOVA test.

Bitter

**Figure 4:**
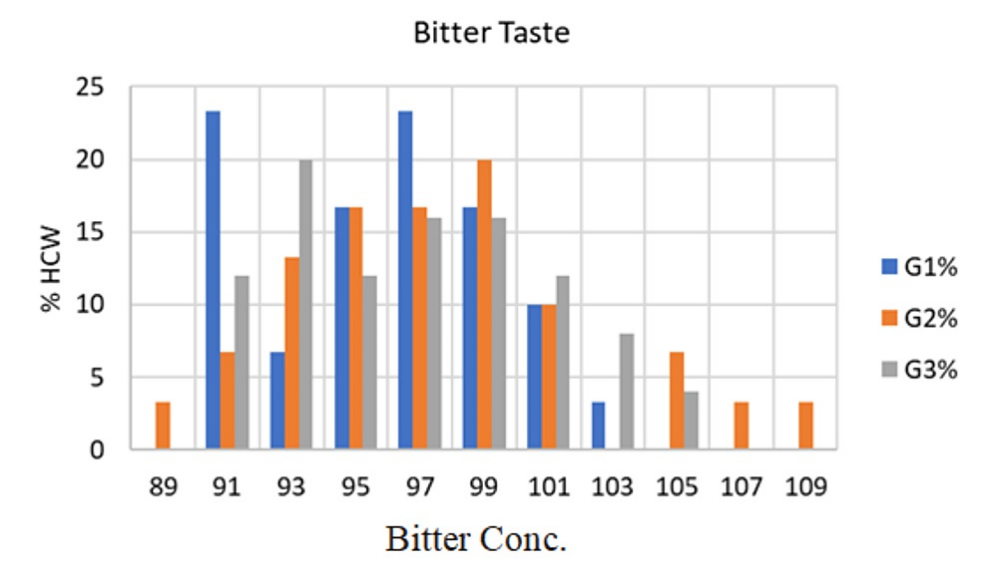
Clinical presentation for taste detection threshold (Bitter) of the three study groups G1: Group 1; G2: Group 2; G3: Group 3; HCW: Health care worker; Conc: concentration

The mean urea concentration detected by subjects in G1 was 95.93±3.59 mmol/L and ranged from 91 to 103 mmol/L. Seven participants (32.3%) tasted bitter at concentration number 2 (91 mmol/L), two participants (6.7%) at concentration number 3 (93 mmol/L), five participants (16.7%) at concentration number 4 (95 mmol/L), and seven participants (23.3%) at concentration number 5 (97 mmol/L). In addition, five participants (16.7%) reported tasting bitterness at concentration number 6 (99 mmol/L), three participants (10.0%) at concentration number 7 (101 mmol/L), and one participant (3.3%) at concentration number 8 (103 mmol/L), as illustrated in Figure 4.

The mean urea concentration detected by subjects in G2 was 97.53±4.75 mmol/L and ranged from 89 to 109 mmol/L. One participant (3.3%) tasted bitter at concentration number 1 (89 mmol/L), two participants (6.7%) at concentration number 2 (91 mmol/L), and four participants (13.3%) at concentration number 3 (93 mmol/L). In addition, five participants (16.7%) tasted bitter at concentration number 4 (95 mmol/L), five participants (16.7%) at concentration number 5 (97 mmol/L), and six participants (20.0%) at concentration number 6 (99 mmol/L). Lastly, three participants (10.0%) reported tasting bitterness at concentration number 7 (101 mmol/L), two participants (6.7%) at concentration number 9 (105 mmol/L), and one participant (3.3%) at concentration number 10 (107 mmol/L), as seen in Figure 4.

The average urea concentration detected by subjects in G3 was 96.84±4.08 mmol/L, with a range of 91 to 105 mmol/L. Three participants (12.0%) reported a bitter taste at concentration number 2 (91 mmol/L), five participants (20.0%) at concentration number 3 (93 mmol/L), and three participants (12.0%) at concentration number 4 (95 mmol/L). In addition, four participants (16%) tasted bitterness at concentration number 5 (97 mmol/L), four participants (16%) at concentration number 6 (99 mmol/L), and three participants (12%) at concentration number 7 (101 mmol/L). Finally, two participants (8.0%) tasted bitterness at concentration number 8 (103 mmol/L), and one participant (4.0%) at concentration number 9 (105 mmol/L).

The taste perception threshold for bitterness was not significantly changed between the means of the concentrations of the three groups (P = 0.335). There was no discernible difference between G1 and G2 (P = 0.147), G2 and G3 (P = 0.568) and between G1 and G3 (P = 0.385) by using the ANOVA test.

## Discussion

Demographic findings (age and gender)

All study participants were chosen from the HCWs. The HCWs are more aware of the possibility of previous COVID-19 infection, which will get more accurate results (as the COVID-19 infection subject is excluded from this study). HCWs consider any flu-like symptoms an infection until a COVID-19 test is carried out. Their ages ranged from 23 to 59 years (young and middle-aged adults) because taste detection thresholds for the elderly (aged adults) increase with age [17].

Taste detection threshold

There was a statistically significant difference in the taste detection threshold of sweets between G1 and G2, as well as G2 and G3. The G2’s sweet taste had a high taste detection threshold, indicating that the vaccine could be one of the causes of decreased sweet flavors detection in subjects after three to six months from the second dose of the Pfizer COVID-19 vaccine.

A literature survey shows that nobody has studied this subject before. However, some studies look at the emergence of smell and taste abnormalities after vaccination, which seems to be a relatively uncommon side effect. This type of research was previously described with the influenza vaccine [18]. No prior studies were found regarding the taste detection thresholds in subjects receiving the Pfizer vaccine.

Despite not observing any gustatory damage, Doty et al. [18] stated that 0.19% of individuals can develop olfactory dysfunction after receiving the influenza vaccine. Mazur et al. [13] found that 2.7% and 3.1% of the sample reported changes in taste sensitivity following the first and second doses. Understanding the pathophysiological mechanisms is still lacking [19]. The most common side effects of vaccination include fatigue, headaches, arthralgia, myalgia, and fever. Studies do not address olfactory or gustatory impairment [20].

Doty et al.'s [18] questionnaire looked into potential taste changes following influenza vaccination. Out of 223 individuals, only three people reported having altered tastes. The questionnaire was not guided by chemosensory testing to determine whether these factors were explicitly connected to the vaccination, general disease, drug intake, the presence/absence of allergies, type 1 diabetes, autoimmune pathologies, or other factors like the severity of the overall adverse vaccination reactions.

The pathophysiological mechanisms are not yet fully understood [20]. However, in the case of SARS-CoV-2 infection, taste impairment is linked to olfactory dysfunction or neurological damage [21]. Although weariness, headaches, arthralgia, myalgia, and fever are among vaccinations’ most frequent side effects, olfactory or gustatory impairment has not been mentioned in research [22].

The study's limitations are that most of the HCWs were already infected with the COVID-19 virus at the time of the study, as they were working in a high viral load environment. So, it was challenging to find never-infected subjects. Taking into account the time of study which was around the time of considering the booster dose to officials in Iraq, people were anxious about taking it. Hence, it was difficult to collect HCWs who were vaccinated with booster dose.

## Conclusions

After three to six months from getting the second dose, the taste detection threshold for sweets was assessed as high. This means the flavor will be harder to perceive. Pfizer COVID-19 immunization may be assumed to be one of the causes of defected taste sensation to sweet flavor. The study test is subjective and cannot simply explain the impairment in taste sensitivity to sweet flavor, hence additional research and analysis on taste detection threshold after Pfizer vaccination is necessary for conclusive results.
